# Ecological Determinants of Sporotrichosis Etiological Agents

**DOI:** 10.3390/jof4030095

**Published:** 2018-08-12

**Authors:** Max C. Ramírez-Soto, Elsa G. Aguilar-Ancori, Andrés Tirado-Sánchez, Alexandro Bonifaz

**Affiliations:** 1 School of Public Health and Administration, Universidad Peruana Cayetano Heredia, Lima 15102, Peru; 2 Fondo Nacional de Desarrollo Científico y Tecnológico y de Innovación Tecnológica (FONDECYT), Consejo Nacional de Ciencia y Tecnologia e Innovacion Tecnologica, Lima 18, Peru; 3 Facultad de Ciencias, Universidad Nacional de San Antonio Abad del Cusco (UNSAAC), Cusco 08003, Peru; elsa.aguilar@unsaac.edu.pe; 4 Instituto Universitario de Enfermedades Tropicales y Biomedicina del Cusco–UNSAAC, Cusco 08003, Peru; 5 Dermatology Service & Mycology Department, Hospital General de México, “Dr. Eduardo Liceaga”, Balmis 148, Colonia Doctores, Ciudad de México 06726, Mexico; atsdermahgm@gmail.com (A.T.-S); a_bonifaz@yahoo.com.mx (A.B.); 6 Internal Medicine Department, Hospital General de Zona 29, Instituto Mexicano del Seguro Social, Ciudad de México 07950, Mexico

**Keywords:** ecological determinants, *Sporothrix* spp., temperature, humidity

## Abstract

Ecological determinants of sporotrichosis etiological agents remain poorly understood. For this reason, we performed explorations using local climate estimates to determine the temperature and humidity ranges of the environment where clinically relevant *Sporothrix* species occur and to identify what plant species are associated with them, using data collected from the published literature. We performed a literature search to identify all publications on environmental isolations of medically relevant species of *Sporothrix* in the PubMed, SCOPUS, and EMBASE databases. All those studies were included in the analysis where medically relevant species of *Sporothrix* have been isolated from soil samples, and described a specific geographical location that could be precisely georeferenced. We approximated temperature and humidity from local climate estimates, integrating geospatial data, temperature, and water vapor pressure from regions or provinces where medically relevant species of *Sporothrix* have been isolated from soil. *Sporothrix* spp. were more commonly isolated from soil of different regions or provinces of 16 countries. Most environmental isolates were identified as *S. schenckii*, whereas *S. pallida*, *S. brasiliensis*, *S. globosa*, and *S. mexicana* were rare. We estimate that medically relevant *Sporothrix* spp. grow in the soil at temperatures of 6.6 °C to 28.84 °C and 37.5% to 99.06% relative humidity. These findings indicate that sporotrichosis etiological agents grow in soil in ecological niches from soil with wide ranges of temperature and humidity, but they are also associated with a variety of plants, flowers, woody debris, reed leaves, corn stalks, leaves, and wood crumbs, potentially facilitating its establishment and proliferation in the environment.

## 1. Introduction

Sporotrichosis is an implantation mycosis caused by the *Sporothrix* spp. [1,2]. It is prevalent in tropical and subtropical areas, and the incidence varies from country to country, with high-prevalence areas located within these endemic focuses [2,3]. *Sporothrix* is a saprophyte of organic matter, dead wood, mosses, hay, and cornstalks [2,4]. It has specific ecological niches within endemic areas, and it grows in soil at a temperature range between 22 °C and 27 °C, pH between 3.5 and 9.4, and 90% humidity [2,3,4,5]. Currently, species groups of *Sporothrix* that are medically relevant include *S. schenckii* (sensu stricto), *S. brasiliensis*, *S. globosa*, *S. luriei, S. mexicana*, and *S*. *pallida* [6,7]. 

Although the geographic distribution of the sporotrichosis is well-characterized [1,3], and it is known that the soil, thorny plants, sphagnum moss, and even certain animals are the reservoirs for sporotrichosis [2,3,4], the association between its etiological agents and ecological factors largely remains uncharacterized. This supports the need to study the possible effects of temperature and humidity of environments where the *Sporothrix* spp. grows. Due to this, we performed explorations using local climate estimates to determine the temperature and humidity ranges of regions where the *Sporothrix* spp. grows, and to identify what plant species were associated with the *Sporothrix* spp., using data collected from the published literature. We focused solely on environmental isolations of medically relevant species of *Sporothrix* (*S. schenckii*, *S. brasiliensis*, *S. globosa*, *S. mexicana*, *S. pallida*, and *S. luriei*).

## 2. Materials and Methods

### 2.1. Literature Search

We performed a literature search of all publications on environmental and soil isolations of *Sporothrix* in the PubMed, SCOPUS, and EMBASE databases up until May 20th, 2018. We also searched the reference lists of the included articles. We applied the Preferred Reporting Items for Systematic Reviews and Meta-Analyses statement (PRISMA) to the methods for this study [8]. Search terms included combinations of the following keywords: environmental, *Sporothrix*, soil, and ecological.

### 2.2. Inclusion and Exclusion Criteria

All studies where medically relevant species of *Sporothrix* have been isolated from soil samples, and which described a specific geographic location that could be georeferenced precisely, were included in the analysis. We excluded studies where the fungus was isolated from a human or animal case, and studies where the geographical boundary of the study area was poorly-defined.

### 2.3. Data Collected

The following data was collected: name of the author(s), year, region, the environment where *Sporothrix* spp. has been isolated, the molecular type of the *Sporothrix* species, and vegetal species associated with the *Sporothrix* fungus.

### 2.4. Statistical Analysis

Since exact coordinates were not specified by the studies where medically relevant species of *Sporothrix* were isolated, latitude, longitude, and elevation coordinates from these regions or provinces were obtained from Google Earth (www.earth.google.com/) by georeferencing [9]. Coordinates for the regions or province were taken based on the centroid of the city, town, or region where samples were taken. To obtain temperature and water vapor pressure (hPa), latitude, longitude, and elevation coordinates obtained from Google Earth were deposited in a software from local climate estimating ‘LocClim 1.0′ (http://www.fao.org/nr/climpag/pub/en0201_en.asp). The distance of the neighboring stations was configured at a distance of at least 500 km. Annual average temperature and water vapor pressure data were obtained after locating the region or province where medically relevant species of *Sporothrix* were isolated. To determine the saturated vapor pressure and relative humidity (%) of the environment, the following equations were used, as described previously [10]:

$$e_{s}=6.11\;\times \;10^{\frac{\left(7.5\times T\right)}{\left(273.3+T\right)}}$$

$$\mathrm{RH}=\frac{e}{e_{s}}\;\times \;100$$

e_s_: saturated steam pressure
T: temperature
e: current steam pressure
RH: relative humidity (%)

## 3. Results

### 3.1. Studies Included

The searches identified 212 records. After 212 titles and abstracts were reviewed, 189 records that did not meet the inclusion criteria were excluded. Finally, 23 studies that reported environmental and soil isolations of medically relevant *Sporothrix* spp. were included [11,12,13,14,15,16,17,18,19,20,21,22,23,24,25,26,27,28,29,30,31,32,33]. 

### 3.2. The Geographical Distribution of Environmental Isolations of Sporothrix *spp*.

**Figure 1:**
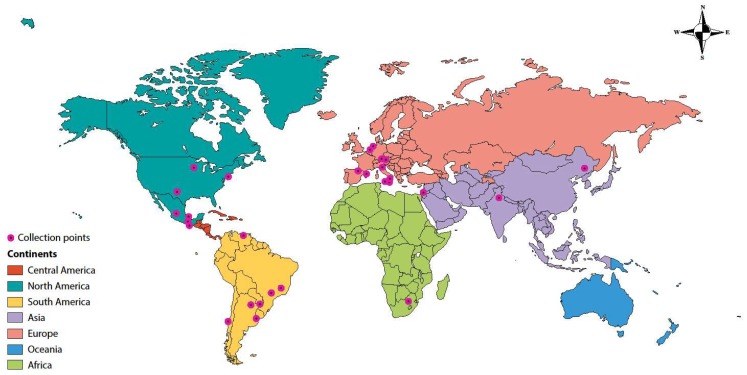
Geographic distribution of environmental isolations of medically relevant *Sporothrix* spp.

*Sporothrix* spp. were isolated from the environment of regions or provinces of Argentina, Austria, Brazil, Chile, China, Germany, India, Israel, Italy, Mexico, Spain, South Africa, Netherlands, United States, Uruguay, and Venezuela. Isolations were most commonly recovered from the provinces of Mexico (Figure 1 and Table 1). 

### 3.3. Sources of Environmental Isolations of Sporothrix *spp*.

In this review, environmental isolates of *Sporothrix* were recovered from different environmental sources. Isolations were mostly obtained from the soil. Other sources were of leaves, flowers, woody debris, reed leaves, old reed leaves, corn stalks, old corn stalks, leaves, and wood crumbs (Table 1).

### 3.4. Identified Sporothrix *spp*.

Six *Sporothrix* spp. were identified. In most reports, identification of *Sporothrix* spp. was carried out using standard phenotypic methods (macroscopic and microscopic characteristics of the fungus). Additional established molecular methods were also used to identify the *Sporothrix* spp. (*S. schenckii s. str.*, *S. pallida*, *S. brasiliensis*, *S. globosa*, *S. chilensis*, and *S. mexicana*). Most environment isolates were identified as *S. schenckii*, whereas *S. pallida* (2 isolates), *S. brasiliensis* (1 isolate), *S. globosa* (1 isolate), *S. chilensis* (1 isolate), and *S. mexicana* (1 isolate) were rare. *S. schenckii* was identified in Argentina, Austria, Brazil, China, Germany, India, Israel, Italy, Spain, Netherlands, United States, Uruguay, and Venezuela. Mexico had the highest number of *S. schenckii* isolates identified. *S. globosa, S. pallida* and *S. chilensis* were isolated in Chile, *S. brasiliensis* in Argentina, *S. mexicana* in South Africa, and *S. pallida* in The Netherlands (Table 1). 

### 3.5. Temperature and Relative Humidity

Since temperature and relative humidity were not specified by the 21 studies where medically relevant species of *Sporothrix* were isolated, we estimated the temperature and relative humidity ranges of the environments where the *Sporothrix* fungus grows. Overall, we estimate that medically relevant *Sporothrix* spp. have specific ecological niches within endemic areas, and they grow in the environment of soil at a temperature between 6.6 °C and 28.84 °C, and relative humidity between 37.5% and 99.06%.

The temperature and relative humidity ranges of the environments where *Sporothrix* fungus grows showed great variations, even in cases where the distributions were within the same country. For example, in Mexico *S. schenckii* grows in the soil at temperatures of 16.23 °C to 28.84 °C and 37.5% to 99.06% relative humidity, whereas in the United States, it grows in the soil at temperatures of 7.63 °C to 18.05 °C and 72.66% to 93.55% relative humidity. In Latin America*, S. schenckii* grows in the soil in Brazil at a temperature of 19.09 °C and 87.88% relative humidity, in Chaco-Argentina *S. brasiliensis* and *S. schenckii s. str*. grow in the soil at a temperature of 20.92 °C and 89.21% relative humidity, in Chile *S. globosa* and *S. pallida* grows in the soil at temperatures of 12.66 °C to 14.76 °C and 85.95% to 87.35% relative humidity, and in Uruguay and Venezuela *S. schenckii s. str.* grows in the soil at temperatures of 17.1 °C and 23.47 °C and 83.48% and 96.62% relative humidity, respectively (Table 1).

In Asia, *S. schenckii* grows in the soil in China at a temperature of 6.6 °C and 78.42% relative humidity, and in India *S. schenckii* grows in the soil at a temperature of 15.48 °C and 68.22% relative humidity. In South Africa, *S. schenckii* and *S. mexicana* grows in the soil at temperatures of 16.15 °C to 18.24 °C and 65.89% to 68.52% relative humidity (Table 1). 

## 4. Discussion

The survival of fungi in their environmental niches depends on their ability to adapt to changing conditions [4]. Isolation of *S. schenckii* from nature has been reported both in endemic and non-endemic areas in various environmental conditions [11,12,13,14,15,16,17,18,19,20,21,22,23,24,25,26,27,28,29,30,31,32,33]. Our findings indicated that *Sporothrix* spp. are isolated mostly from soil, but they are also associated with a variety of plants, flowers, woody debris, reed leaves, corn stalks, leaves, and wood crumbs [11,12,13,14,15,16,17,18,19,20,21,22,23,24,25,26,27,28,29,30,31,32,33], potentially facilitating its establishment and proliferation in the environment. Therefore, it will take advantage of the opportunity to infect a mammal host, including humans, cats, and dogs [34]. Sporotrichosis is often referred to as ‘rose handler’s disease’, since the fungus has long been thought to be acquired by means of trauma associated with rose bush spines and other plant materials as Sphagnum moss [1,2,3]. The sprouts responsible for propagation or vegetative reproduction in plants, also called propagules, usually present in the state of nature, are the main source of infection for the patients with sporotrichosis, through a traumatic inoculation in Tropical and Subtropical zones. The soil constitutes another reservoir of the fungus, as well as some insects like beetles and ants [13]. 

Although the geographical distribution of clinically-relevant *Sporothrix* species has been intensively studied, these fungi have been hard to isolate from the environment probably due to their low concentration in environmental samples. In addition, *Sporothrix* spp. are slow-growing fungi, and on rich culture media, their colonies are easily obscured by fast growing molds such as *Penicillium*, *Aspergillus*, or Mucorales [35]. In the present study, six *Sporothrix* spp. were identified as *S. schenckii s. str*., *S. pallida*, *S. brasiliensis*, *S. globosa, S. chilensis*, and *S. mexicana*. The different species of the so-called *Sporothrix schenckii* complex are environmental fungi found in soils, plants, water, decaying plants, and other outdoor environments [4,5]. Although they have been isolated from diverse environments, few studies have pointed to the influence of the environment (temperature and humidity) on the virulence of these pathogens. However, some research in *S. schenckii* and other cryptic species suggest that adverse conditions in the natural habitat may trigger the expression of different virulence factors, conferring survival advantages both in the environment and in the host tissue [4].

Environmental microorganisms are usually exposed to different physical factors, such as extreme temperatures, salinity, sunlight (in direct relation with latitude), and drought. *S. schenckii* is able to withstand extreme conditions, such as very low temperatures [36,37] and extreme osmotic pressure [2,38] for several years. Similarly, there is evidence that *S. schenckii* is able to resist the influence of solar radiation. The exposure of *S. schenckii* to different levels of UV light results in a conserved viability [39,40]. The environmental stressor promotes the virulence of *S. schenckii*; the origin of virulence in *S. schenckii* must be related to the interactions of the pathogen with the different environmental challenges present in its natural habitat, such as extreme temperatures (highs and lows), as we observed in our study, where the isolation was made in geographical areas with extreme temperatures, humidity/drought, radiation, and chemical contamination. These environmental stressors are tolerated due to constitutive fungal structures and inducible molecules acquiring survival capacity and becoming virulence factors in the infected host. The interaction of dimorphic fungi with a mammalian host is not a requirement for the survival and virulence of fungi, as in the case with other pathogenic microorganisms [39]. This phenomenon is called “ready-to-use” virulence [40] and the environment may contribute to the origin and maintenance of virulence in certain fungi [41,42,43,44,45,46]. One of the virulence factors of *Sporothrix* spp. is their ability to convert to yeast phase when they enter the human body. However, a non-clinical species, *‘Ophiostoma´ bragantinum* with a *Sporothrix* asexual morph, is able to convert into yeast cells when it is grown at temperatures near 30 °C. The ability to form yeast cells might be widely present in the genus *Sporothrix* [47]. Although many external influences are known to affect the pathogenicity of the *S. schenckii* complex, these influences and mechanisms have not been sufficiently studied. However, the existence of common molecules that interact with environmental stressors, described in various environmental fungi, leads us to the hypothesis that similar mechanisms may be acting in *S. schenckii*, regarding their adaptation to these extreme conditions [4]. Several virulence factors can be produced by fungi to survive both in animal hosts and in the environment. This phenomenon is called “dual use” [48]. Understanding the interactions between fungi and their potential hosts in the environment is in its initial stage. However, initial observations suggest that this will be an extremely rich area of research to further explore fundamental issues of fungal pathogenesis [49,50]. 

The limitations of our study are the relatively few published reports and methods used. Regarding the former, only 23 studies of 16 countries were included, since published reports on *Sporothrix* show that clinical and veterinary isolations have always outnumbered environmental isolations. Regarding the latter, according to the methods used to estimate the temperature and humidity ranges of environments where the *Sporothrix* fungus grows, our findings only provide an approximate estimate of temperature and humidity ranges in certain regions of the countries studied. However, these data can also constitute gross underestimations or overestimations of the true temperature and the humidity of the environment where the *Sporothrix* spp. grows. Finally, several apparently non-pathogenic species (e.g., *S. brunneoviolacea*, *S. dimorphospora* and *S. inflata*) are morphologically very similar to *S. schenckii* and its pathogenic relatives. Therefore, reports of isolations not supported by DNA sequence data may sometimes represent misidentifications. Despite these limitations, our findings may contribute to the design of new strategies for the control of sporotrichosis in the future.

## 5. Conclusions

Although limited, the results of our study indicate that sporotrichosis etiological agents grow in soil ecological niches from soil with wide ranges in temperature and humidity, but they are also associated with a variety of plants, flowers, woody debris, reed leaves, corn stalks, leaves, and wood crumbs, potentially facilitating its establishment and proliferation in the environment. Therefore, more studies that evaluate the influence of different environmental factors on the physiology and pathogenicity of the *S. schenckii* complex are necessary, although all available data suggests the existence of strategies that pathogenic fungi acquire to survive adverse environmental conditions. In turn, these mechanisms of acquired resistance provide the fungi with the ability to infect animals and can allow the emergence of opportunistic pathogens from these microenvironments [49].

## Figures and Tables

**Table 1:** Studies overview environmental and soil isolations of medically relevant *Sporothrix* spp.

Country	Ref.	Collection Year	Region	Molecular Identification	Source	Species	Longitude *	Latitude *	Elevation (Meters) *	Temperature (°C) *	RH (%) *
Argentina	[11]	2003	Chaco	*S. brasiliensis*	Soil	*S. brasiliensis*	−59.018371°	−27.420760°	53	20.92	89.21
		2004	Chaco	*S. schenckii*	Soil	*S. schenckii*					
		2001	Misiones	*S. schenckii*	Soil	*S. schenckii*	−55.960100°	−27.443478°	153	21.14	89.15
Austria	[12]	2007–2008	Salzburg, Austria	*S. schenckii*	Garden soil	*S. schenckii*	13.051580°	47.810801°	429	8.91	86.91
Brazil	[13]	2014	Botucato, Sauñe Paulo	*S. schenckii (sensu stricto)*	Armadillo soil	*S. schenckii* (*sensu stricto*)	−48.437236°	−22.892863°	793	19.19	87.88
	[14]	1963	Piracicaba and Vicinity	…	Soil	*S. schenckii*	−43.905655°	−19.926731°	904	19.54	95.97
Chile	[15]	2012	Valparaiso	…	Soil	*S. globosa*	−71.595196°	−33.094955°	460	12.66	87.35
	[16]	2014	Viña del Mar	*S. pallida*	Garden soil	*S. pallida*	−71.549622°	−33.012123°	17	14.76	85.95
	[17]	2016	Viña del Mar	*S. chilensis*	Garden soil	*S. chilensis*					
China	[18]	2002	Jilin	*S. schenckii*	Soil, reed leaves, old reed leaves, corn stalks and old corn stalks, Cettal leaves, and wood crumbs	*S. schenckii*	126.414149°	43.144094°	521	6.60	78.42
Germany	[12]	2007–2008	Rain Am Lech, Germany	*S. schenckii*	Amended soil	*S. schenckii*	10.922538°	48.690341°	412	8.24	91.41
India	[19]	2007	Himachal Pradesh	…	Corn stalk	*S. schenckii*	77.191581°	31.113026°	1858	15.48	68.22
Israel	[20]	1976	Petah Tiqua		Soil	*S. schenckii*	34.887816°	32.083751°	57	19.35	79.73
Italy	[12]	2007–2008	Reggio Calabria	*S. schenckii*	Natural soil	*S. schenckii*	15.661199°	38.102348°	62	18.01	84.86
			Serra San Bruno	*S. schenckii*	*Sphagnum moss*	*S. schenckii*	16.314224°	38.562623°	830	12.14	86.58
			Reggio Calabria	*S. schenckii*	Amended soil	*S. schenckii*	15.638878°	38.104305°	8	18.42	84.6
			Verona	*S. schenckii*	Garden soil	*S. schenckii*	10.994555°	45.448563°	59	13.29	86.19
			Calabria	*S. schenckii*	Enviromental	*S. schenckii*	16.349470°	39.312534°	857	11.91	83.69
			Sicily	*S. schenckii*	Enviromental	*S. schenckii*	14.038756°	37.598328°	507	15.38	81.74
	[21]	2007	Huauchinango-Puebla	…	Soil associated with pine, rose, gladiolus and wild plant	*S. schenckii*	−98.062507°	20.175399°	1553	16.23	99.06
	[22]	2004	Santa Maria quielogani, Oaxaca	…	Farmland	*S. schenckii*	−96.056315°	16.278295°	2061	28.84	37.5
Mexico	[23]	2017	Atlixco	…	Soil	*S. schenckii*	−98.429715°	18.911546°	1842	18.74	78.34
			Cholula	…	Soil	*S. schenckii*	−98.300998°	19.076175°	2168	16.79	73.49
			Izúcar de Matamoros	…	Soil	*S. schenckii*	−98.464283°	18.598513°	1285	22.95	83.63
			Tecali de Herrera	…	Soil	*S. schenckii*	−97.974147°	18.901923°	2175	16.88	72.18
			Tecamachalco	…	Soil	*S. schenckii*	−97.733545°	18.879810°	2015	18.23	70.32
			Tehuacan	…	Soil	*S. schenckii*	−97.393711°	18.466471°	1640	19.04	79.37
			Tepexi de Rodriguez	…	Soil	*S. schenckii*	−97.928542°	18.584326°	1690	21.02	72.53
			Atlixco	…	Soil associated with *Eucalyptus camaldulensis*, *Rosa centifolia*, *Zea mays*	*S. schenckii*	−98.437736°	18.912245°	1940	17.96	78.22
Mexico	[23]	2017	Izúcar de Matamoros	…	*Curcurbita* sp., *Stenocereus marginatus*, and *Cupressus lindleyi*	*S. schenckii*	−98.461786°	18.579067°	1266	23.23	79.85
			Puebla	…	*Jacaranda mimosaefalia* and *Oreodoxa regia* (palma)	*S. schenckii*	−98.207416°	19.047159°	2163	17.00	75.18
			Tecali de Herrera	…	Plants	*S. schenckii*	−97.973964°	18.914850°	2186	16.79	72.11
			Tecamachalco	…	Plants	*S. schenckii*	−97.749386°	18.882578°	2015	19.39	66.48
			Tehuacan	…	Plants	*S. schenckii*	−97.371094°	18.465983°	1562	19.37	80.43
			Tepexi de Rodriguez	…	Plants	*S. schenckii*	−97.920730°	18.585191°	1655	21.27	72.53
Mexico			Guadalajara	…	Brachiaria plataginea	*S. schenckii*	−103.442383°	20.595692°	1627	19.33	69.28
	[24]	1998	Tonala	…	Sandy soil, sandy soil with organic matter, moderately sandy soil, and clay soil	*S. schenckii*	−103.230673°	20.622654°	1637	19.52	69.2
			Zapopan	…	Soil	*S. schenckii*	−103.415036°	20.671554°	1626	19.30	68.83
	[25]	2009	Huachinango Puebla	*S. schenckii*	Soil	*S. schenckii*	−98.062507°	20.175399°	1553	16.23	99.06
			Xilocuautla Puebla	*S. schenckii*	Soil	*S. schenckii*	−98.023551°	20.138984°	1630	15.50	98.85
			San Andrés Larraizar Chiapas	*S. schenckii*	Soil	*S. schenckii*	−92.712955°	16.882654°	2220	16.54	72.09
Spain	[12]	2007–2008	Barcelona	*S. schenckii*	Amended soil	*S. schenckii*	2.185471°	41.387742°	8	16.85	81.33
		2007–2008	Navarra	*S. schenckii*	Garden soil	*S. schenckii*	−1.652109°	42.817402°	442	12.42	79.29
South Africa	[26]	1986	Pretoria	…	Soil	*S. schenckii*	28.184889°	−25.742540°	1311	18.24	65.89
	[27]	2014	South Africa	*S. mexicana*	Soil of mines	*S. mexicana*	28.070048°	−26.229788°	1684	16.15	68.52
The Netherlands	[28]	2008	Centraalbureau voor Schimmelcultures, Utrecht	*S. pallida*	Garden soil	*S. pallida*	5.176516°	52.089437°	0	9.44	93.98
	[12]	2007–2008	Vriezenveen	*S. schenckii*	Amended soil	*S. schenckii*	6.614617°	52.408313°	11	9.14	95.32
United States	[29]	1970	Winsconsisn	…	Soil, wood, sphagnum were assayed for pathogenic fungi	*S. schenckii*	−88.492690°	43.767366°	244	7.63	93.55
	[30]	1988	New York	…	*Sphagnum moss*	*S. schenckii*	−73.978368°	40.732011°	3	12.67	82.41
	[31]	1984	Texas	…	Potting soil	*S. schenckii*	−99.955168°	31.958474°	558	18.05	72.66
Uruguay	[32]	1969	Montevideo	…	Soil and plant remains, armadillo soil, moss floors, dry grassland of armadillo nest	*S. schenckii*	−56.207668°	−34.859734°	12	17.10	83.48
Venezuela	[33]	2007	Estado Aragua-Caracas	…	Land with and without fertilizer	*S. schenckii*	−67.278619°	10.231817°	547	23.47	96.62

* Coordinates, temperature, and relative humidity obtained in the present study. RH: relative humidity.
